# The application of a heat‐inducible CRISPR/Cas12b (C2c1) genome editing system in tetraploid cotton (*G. hirsutum*) plants

**DOI:** 10.1111/pbi.13417

**Published:** 2020-06-08

**Authors:** Qiongqiong Wang, Muna Alariqi, Fuqiu Wang, Bo Li, Xiao Ding, Hangping Rui, Yajun Li, Zhongping Xu, Lei Qin, Lin Sun, Jianying Li, Jiawei Zou, Keith Lindsey, Xianlong Zhang, Shuangxia Jin

**Affiliations:** ^1^ National Key Laboratory of Crop Genetic Improvement Huazhong Agricultural University Wuhan China; ^2^ Department of Biosciences Durham University Durham UK

**Keywords:** plant, cotton (*G. hirsutum*), CRISPR/Cas12b, heat‐inducible genome editing, off‐target effects

## Abstract

The CRISPR/Cas9 and Cas12a (Cpf1) tools have been used on a large scale for genome editing. A new effector with a single nuclease domain, a relatively small size, low‐frequency off‐target effects and cleavage capability under high temperature has been recently established and designated CRISPR/Cas12b (C2c1). Cas12b has also shown temperature inducibility in mammalian systems. Therefore, this system is potentially valuable for editing the genomes of plant species, such as cotton, that are resistant to high temperatures. Using this new system, mutants of upland cotton were successfully generated following *Agrobacterium*‐mediated genetic transformation under a range of temperatures. Transformants (explants infected by *Agrobacterium*) exposed to 45 °C for 4 days showed the highest editing efficiency. No off‐target mutation was detected by whole‐genome sequencing. Genome edits by AacCas12b in T0 generation were faithfully passed to the T1 generation. Taken together, CRISPR/Cas12b is therefore an efficient and precise tool for genome editing in cotton plants.

## Introduction

The diversity, modularity and efficacy of CRISPR‐Cas systems have led to a biotechnological revolution (Jia and Wang, 2020; Knott and Doudna, 2018; Van Vu *et al*., 2020). Currently, the CRISPR/Cas9 system has become the most widely used genome editing tool, in which a complex composed of crRNA and tracrRNA with Cas9 protein has a multifunctional engineering ability, such as for gene knockout, gene knockdown and base editing (Cho *et al*., 2013; Li *et al*., 2019b; Li *et al*., 2019a; Qin *et al*., 2020; Tang *et al*., 2017). This editing system can identify specific genomic sequences efficiently, with a huge potential for understanding gene function (Jinek *et al*., 2012; Mali *et al*., 2013; Ran *et al*., 2015). However, there are some limitations of the CRISPR/Cas9 system, including off‐target effects, difficulties in PAM sequence selection for fewer potential target sites, and difficulties in generating homozygous mutations in the offspring (Pattanayak *et al*., 2013; Ran *et al*., 2013; Zeng *et al*., 2020). Therefore, there is interest in modifications to the CRISPR/Cas9 system to reduce off‐target effects, improve the accuracy of gene editing, and expand the scope of targeting and in finding new gene editing systems (Haeussler and Concordet, 2016; Manghwar *et al*., 2020; Manghwar *et al*., 2019).

Cas12b (C2c1) is a type V‐B dual‐RNA‐guided endonuclease belonged to class 2 CRISPR‐Cas. Its distinctive features make it unique than other members of class 2 CRISPR‐Cas systems, such as Cas12a and Cas9 (Liu *et al*., 2017). The AacCas12b is derived from *Alicyclobacillus acidoterrestris*, with 20 nt target length and has a PAM sequence of 5'‐TTN‐3' located upstream the target site (Shmakov *et al*., 2015). The chimeric single guide RNA (sgRNA), formed by base pairing of crRNA and tracrRNA and involved in genome editing, needs to combine with Cas12b protein to form a complex, and then combines with the target DNA sequence to complete the cleavage (Liu *et al*., 2017; Yang *et al*., 2016). DNA cleavage occurs at the 23 bases downstream the non‐target chain PAM region and between bases 14 and the 17 of the targeted strand (Liu *et al*., 2017; Wu *et al*., 2017). Therefore, Cas12b can produce DNA double‐stranded breaks with 6‐8 nt sticky ends, representing the longest sticky ends of all current CRISPR‐Cas systems, and promote non‐homologous recombination repair. The CRIPSR/ Cas12b system has been shown to cleave the targeted genome in mammals (Teng *et al*., 2018). More importantly, this system is highly sensitive to mismatches (Strecker *et al*., 2019). Just one single nucleotide mutation in the first 18 nucleotides of the target sequence is sufficient to prevent the cleavage process (Liu *et al*., 2017). Mismatches at the latter two nucleotides can reduce its activity. Therefore, the CRISPR/Cas12b system potentially has the lowest off‐target rate for gene editing and so could be a better alternative for therapeutic and clinical applications (Jain *et al*., 2019). On the other hand, CRISPR/Cas12b is a heat‐induced system which requires a temperature ranging between 40 and 55 °C for effective cleavage, when the temperature is lower than 40 °C, cleavage cannot be accomplished (Liu *et al*., 2017; Shmakov *et al*., 2015; Yang *et al*., 2016). As more variants of Cas12b are explored, the adaptation temperature and its efficiency are constantly being optimized (Teng *et al*., 2019). CRISPR/Cas9 and CRISPR/Cas12a have been widely adopted for plant genome editing, but Cas12b has not been widely studied (Zhang *et al*., 2019). Therefore, it would be of great value to apply this new system in thermophilic crop species like cotton.

Cotton (*Gossypium hirsutum*) is allotetraploid, following the ancestral hybridization of two diploid genomes (A and D). Almost all genes have multiple copies, with few differences in their sequences, that make the cotton genome complex and hard to manipulate by mutagenesis. Precise site‐specific editing tools offer a solution. Cotton is also a thermophilic plant species that can survive under temperatures above 40 °C at the end of July in most cotton cultivation regions (Ekinci *et al*., 2017). Therefore, the feasibility of Cas 12b system from *Alicyclobacillus acidoterrestris* (AacCas12b) is tested for cotton genome editing for the first time considering the thermophilic habit of cotton plants and higher activity of AacCas12b under the temperature is above 40 °C. This report provides some interesting features of AacCas12b system, such as the temperature‐related activity and undetectable off‐target effects, which could be applied to assistant better application of genome editing induced by the Cas12b systems in plants.

## Results and discussion

### CRISPR/Cas12b system‐induced targeted mutations

**Figure 1:**
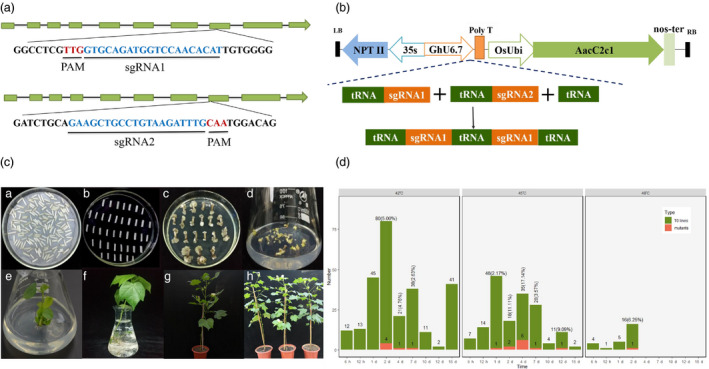
Vector, sgRNAs map, genetic transformation and generated plant through AacCas12b system in cotton. (a) Schematic view of sgRNA1 and sgRNA2 target sites in the *GhCLA* gene. The target sequences are highlighted in blue, and the PAM sites are highlighted in red. (b) Schematic of the T‐DNA region of GhRCas12b vector. (c) The *Agrobacterium*‐mediated genetic transformation and plant regeneration of transgenic plants. (c‐A) Co‐culture stage. (c‐B‐C) Callus induction and differentiation. (c‐D) Somatic embryogenesis. (c‐E) Plant regeneration. (c‐F) The acclimatization of regenerated plant in nutrient solution. (c‐G‐H) Transgenic plants grown in the greenhouse. (d) Total number of T0 generated plants and the number of positive edits produced under different temperature conditions.

To investigate whether CRISPR/Cas12b can induce genome editing in plants, two sgRNAs with 20 nt target sequences were designed to induce targeted mutations in the endogenous *CLOROPLASTOS ALTERADOS* (deoxyxylulose‐5‐phosphate synthase; *GhCLA*) gene in cotton (Wang *et al*., 2018). Therefore, we designed the sgRNA and a vector named pRGEB32‐AacCas12b‐GhCLA targeting *GhCLA* gene (Figure 1a,b). Hundreds of T0 individual transgenic lines were obtained through *Agrobacterium*‐mediated genetic transformation and tissue culture for plant regeneration, according to our previous protocol and the detail protocol was illustrated in Figure 1c. The detail information for the heat treatment is shown as follows: 21‐day‐old hypocotyl cuttings infected by *Agrobacterium* for 2 days (harbouring the transformed cells) were subjected to different temperatures treatment at the callus induction stage (Li *et al*., 2019c). A total of 263, 165 and 26 plants were generated under three temperature treatments: 42, 45 and 48 °C, respectively, with eight incubation times (6 h, 12 h, 1 day, 2 days, 4 days, 7 days, 10 days, 12 days and 15 days; Figure 1d).

**Figure 2:**
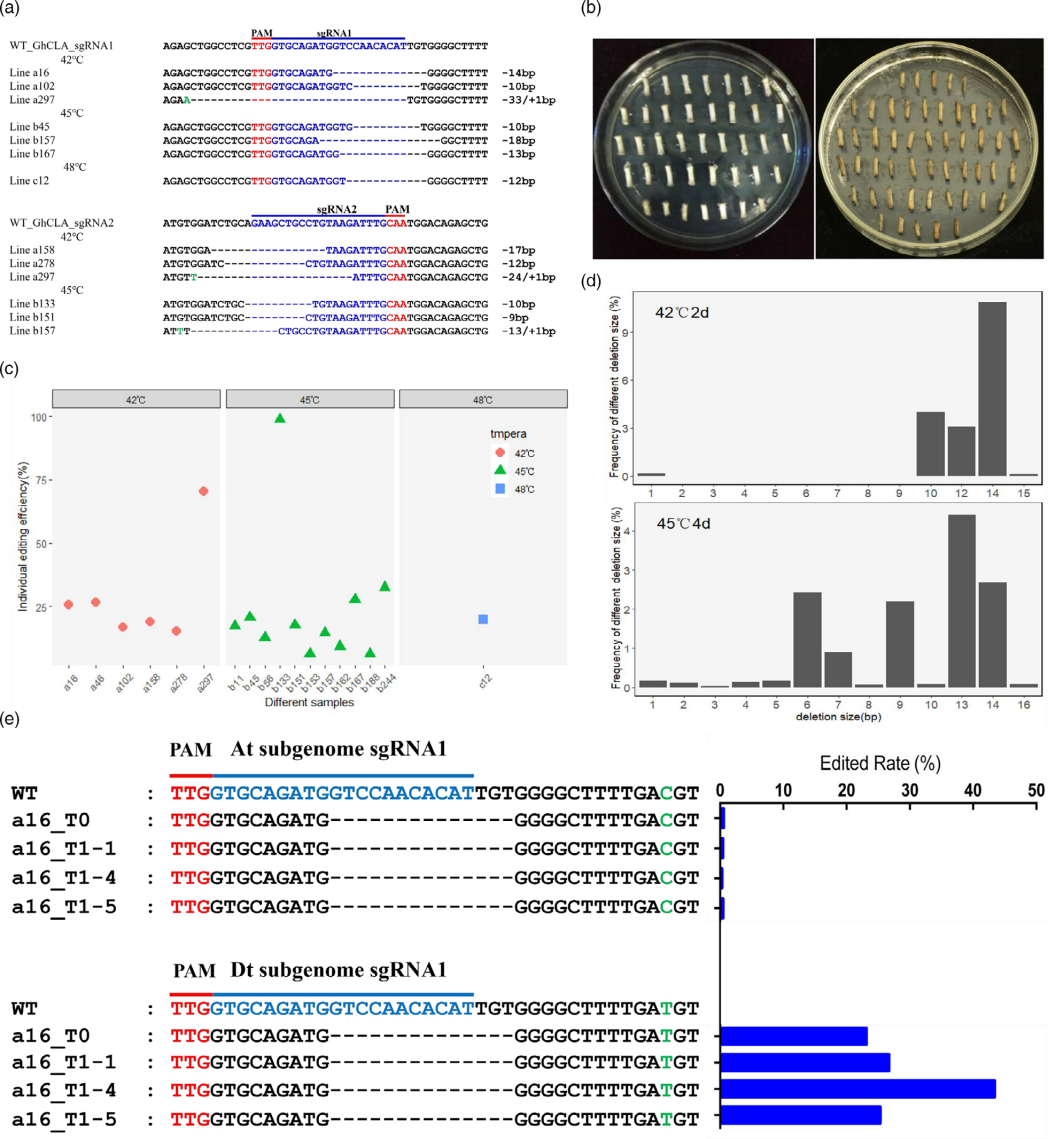
The CRISPR/Cas12b editing in the target sites of cotton genome. (a) AacCas12b‐CLA‐sgRNA1 and AacCas12b‐CLA‐sgRNA2 editing results. PAM in red letters, target sequences in blue letters and substituted in green letters. (b) Hypocotyls without heat treatment after transformation (left) and hypocotyls treated at 48 °C for 4 days after transformation (right). (c) Target mutation extent of T0 plants edited by cotton CRISPR/Cas12b system. The mutation frequency of the *GhCLA* gene in the independent T0 plant. (d) Frequency of different deletion sizes at the target sites of *GhCLA* at 42 °C for 2 days and 45 °C for 4 days. (e) Genotypes revealed from 16 lines of T0 plants to T1 generation plants. The PAM sites are highlighted in red, and crRNA sequence sites are highlighted in blue. The difference between the bases of At and Dt sub‐genome is indicated by the green letters. The histogram on the right indicates the target mutation ratio (the reads with target mutations/ total reads of the target site) from Hi‐tom analysis.

At 42 °C, the 263 transgenic plants were created, of which 6 plants produced predictable edits, distributed at 2, 4 and 7 days, with edit rates of 5.00%, 4.76% and 2.63%. Similarly, at 45 °C, there are 165 transgenic plants were created, and 11 of them showed target editing, and their editing rates were 2.18%, 11.11%, 17.14%, 3.57% and 9.09%, respectively, with 1‐, 2‐, 4‐, 7‐ and 12‐day incubation times. Most mutations occurred at one specific site, from sgRNA1 or sgRNA2 target site; and only one plant showed editing simultaneously from both sgRNA1 and sgRNA2 targets sites with the treatment at 42 °C for 4 days. Most edited types are deletions of DNA fragments in edited plant (Figure 2a). Of the 26 regenerated T0 plants after treatment at 48 °C for 2 days, 1 plant generated a deletion with the size of 12 bp (Figure 1d). All the hypocotyls cutting treated at 48 °C for more than 2 days (>2 days) died, suggesting that 48 °C is the limiting temperature for cotton cell, tissue cultural in vitro (Figure 2b).

Hi‐TOM sequencing data revealed that each individual plant contained diverse editing events. Individuals with a target mutation ratio lower than 1% (calculated as number of reads with target mutations/total reads of the target site) were considered as negative (no editing at the target site), whereas those samples with mutation ratios >1% (i.e. mutations occurred at the target site) were considered as edited. Based on this criterion, the editing frequency in the 18 independent edited plants ranged from 6.34% to 98.68% (Figure 2c). However, the mutation rate was typically about 20% (Figure 2c). Deletion size and proportion of mutations in T0 plants showing the highest editing efficiency, at 42 °C for 2 days and 45 °C for 4 days, were also analysed (Figure 2d). The deletion size ranged from 1 to 16 bp in length, with the majority ranging from 9 to 14 bp (Figure 2d). This is larger than the average deletion size (1–5 bp) reported to be induced by CRISPR/Cas9 in cotton (Wang *et al*., 2018). Our data suggest that the CRISPR/Cas12b system preferentially generates relatively larger DNA fragment deletions in the cotton genome.

The CRISPR/Cas12b system successfully introduced editing in cotton plants under the three temperature treatments. Most plants generated larger DNA fragments deletions, also with some base substitutions (Figure 2a). Incubation at 45 °C for 4 days of the explants resulting in the highest rate of editing and little adverse effect on the differentiation and survival of the callus (Figure 1d), indicating this is the optimum temperature for cotton genome editing. Incubation periods of <2 days at any of the three temperature treatments resulted in either no editing or a low editing efficiency. The temperature used in this study (42 °C) led to the lowest editing efficiency. Although 45 °C promoted efficient editing, callus maintained at this temperature for prolonged periods remained at the differentiation stage and was unable to develop to the re‐differentiation stage or died. A 4 days processing time had no significant adverse effect on the cell differentiation and survival of the callus. Although editing efficiency increases with temperature, the adverse effects on cell viability can limit usefulness. For example, hypocotyls died after 2‐day incubation at 48 °C (Figure 2b). In summary, to achieve efficient cleavage activity of CRISPR/Cas12b system, we suggest that 45 °C for 4‐day incubation time is the optimum condition to cotton cell culture and genome editing generated by AacCas12b.

The heritability of editing events from the T0 plants (a16 line) to the T1 generation was investigated. Results showed that the mutated genotype was faithfully inherited from T0 to T1 generation (Figure 2e). Interestingly, the editing efficiency for four plants at the genome sites of Dt sub‐genome was obviously higher than those sites at the At sub‐genome suggesting that CRISPR/Cas12b might preferentially edit the Dt cotton sub‐genome rather than the At sub‐genome due to chromatin structure or some unknown reasons.

### Whole‐genome sequencing analysis revealed minimal off‐target effects in cotton

**Figure 3:**
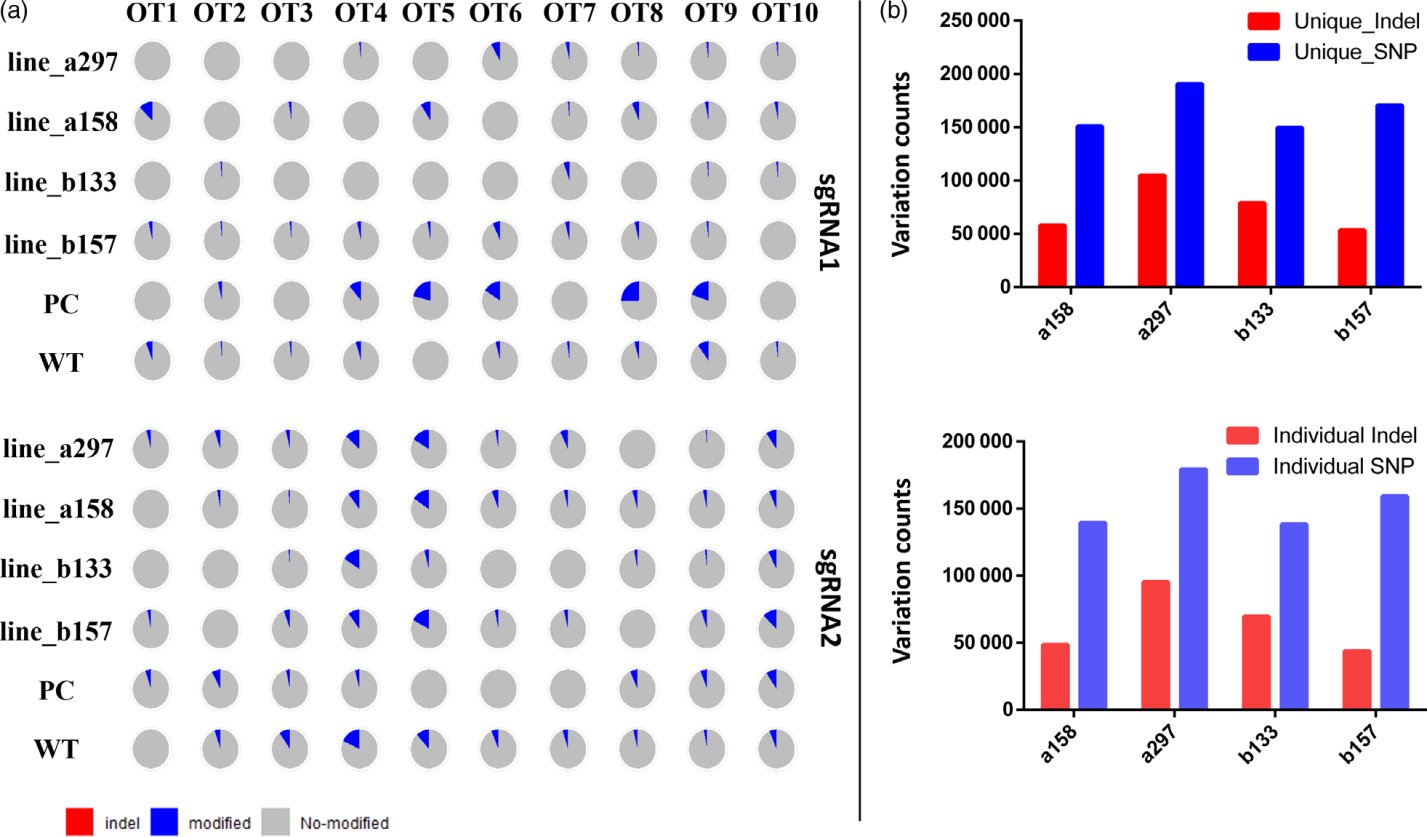
The evaluation of off‐target effects in AacCas12b‐edited cotton plants by whole‐genome sequencing (WGS). (a) The mutation patterns were detected in 10 most potential off‐target sites between sgRNA1 and sgRNA2. Every pie chart represents one off‐target mutation for one plant. (b) The up and the bottom panels represent the sgRNA1 and sgRNA2 variations, respectively.

Finally, we assessed AacCas12b off‐target effects in cotton plants on a genome‐wide scale. Whole‐genome sequencing (WGS) with 50× sequencing depth was performed on four edited plants (lines a158, a297, b133, b157), one wild type (WT) and one positive control (PC) plant (generated through tissue culture and containing a T‐DNA insertion with CRISPR‐Cas12b sequence without temperature induction) (Li *et al*., 2019b). Total of 1864 and 1490 potential off‐target sites was detected for sgRNA1 and sgRNA2, respectively. The ten most likely off‐target sites (OT1‐OT10) were selected for each sgRNA (Table 1) and compared with the four edited plants, the WT and the PC plants (Table 2). Sequencing data indicated that off‐target mutations for both sgRNAs were very low (<10%), and no indel was detected (Figure 3a).

**Table 1:** The most ten potential off‐target sites calculated by the Cas‐OFFinder software

sgRNA	Sequence	ID
sgRNA1	TTGGTGCAGATGGaCCAACACAT	OT1
	TTGGTGCAGATGGaCCAACgCAT	OT2
	TTCcTGCAGATGGgCCAACACtT	OT3
	TTAGTGCtGATGGTCCAAttCAT	OT4
	TTTtTGCAGATGGgCtAACACAT	OT5
	TTTtTGCAGATGGgCtAACACAT	OT6
	TTTGTGCAGATtGTggAAgACAT	OT7
	TTTGTcCAcATGGgCaAACACAT	OT8
	TTTGTGCAcATGGgCagACACAT	OT9
	TTGGTGCAcATGagCtAACACAT	OT10
sgRNA2	TTTCAAATCTTACAGGCAGCTaC	OT1
	TTTCAAATCTTACAGaCAGCTaC	OT2
	TTTCAAATCTTACAGaCAGCTaC	OT3
	TTTCAAATCTTACAGaCAGCTaC	OT4
	TTTCAAATCTTACAGaCAGCTaC	OT5
	TTTCAAATCTTAgtGGCtGCTTC	OT6
	TTGCAAATCTTACtGGtAGCTTt	OT7
	TTGCAAATCTcACAGGgAGCTTt	OT8
	TTACAAATCTTtCAGGaAaCTTC	OT9
	TTTCAAATtTTACAGaCAGCTaC	OT10

Sequence: sequence of potential off‐target sites with lowercase letters representing mismatches.

**Table 2:** Detection of the potential off‐target sites by whole‐genome sequencing

Lines	sgRNA	Mutation	Mutation rate (%)									
			OT1	OT2	OT3	OT4	OT5	OT6	OT7	OT8	OT9	OT10
a297	sgRNA1	Indel	0	0	0	0	0	0	0	0	0	0
		Modified	0	0	0	1.82	0	9.09	3.85	1.75	2.22	1.72
	sgRNA2	Indel	0	0	0	0	0	0	0	0	0	0
		Modified	4.26	5.56	3.90	15.31	19.51	2.86	7.69	0	1.15	10.53
a158	sgRNA1	Indel	0	0	0	0	0	0	0	0	0	0
		Modified	14.29	0	2.70	0	10	0	1.64	7.14	3.45	3.33
	sgRNA2	Indel	0	0	0	0	0	0	0	0	0	0
		Modified	0	2.94	1.43	11.69	18.75	6.45	3.85	5.00	3.77	7.14
b133	sgRNA1	Indel	0	0	0	0	0	0	0	0	0	0
		Modified	0	1.64	0	0	0	0	5.77	0	1.59	1.85
	sgRNA2	Indel	0	0	0	0	0	0	0	0	0	0
		Modified	0	0	1.22	18.99	4.35	0	0	3.17	1.89	8.11
b157	sgRNA1	Indel	0	0	0	0	0	0	0	0	0	0
		Modified	3.75	1.54	1.82	3.77	2.90	7.14	4.35	4.44	2.00	0
	sgRNA2	Indel	0	0	0	0	0	0	0	0	0	0
		Modified	3.33	0	5.77	11.49	20.83	3.45	3.80	0	5.61	14.71
PC	sgRNA1	Indel	0	0	0	0	0	0	0	0	0	0
		Modified	0	3.85	0	12.50	26.67	18.75	0	34.21	25.00	0
	sgRNA2	Indel	0	0	0	0	0	0	0	0	0	0
		Modified	5.26	8.57	3.70	4.26	0	0	0	7.14	6.38	10.53
WT	sgRNA1	Indel	0	0	0	0	0	0	0	0	0	0
		Modified	6.25	1.49	2.04	5.00	0	4.17	2.53	4.35	11.32	2.13
	sgRNA2	Indel	0	0	0	0	0	0	0	0	0	0
		Modified	0	5.56	10.71	22.41	13.33	6.67	5.13	3.92	3.03	6.67

Modified: base substitution mutations; Indel: delete and insertion.

To further evaluate potential off‐target mutations caused by AacCas12b, two variant caller tools with strict parameters were applied to obtain high concordance variations. For line a158, a total of 1 773 469 indels and 3 237 186 SNPs were detected compared with the TM‐1 reference genome; for a297, there were 1 772 644 indels and 3 237 274 SNPs; for b133, 1 772 789 indels and 3 237 410 SNPs; for b157, 1 773 068 indels and 3 237 395 SNPs; for PC, 773 889 indels and 3 237 136 SNPs; and for WT, 1 774 432 indels and 3 23 453 SNPs (Table 3). We also used data from one negative control plant and one WT plant from a previous study as a control for the evaluation of somaclonal variation following tissue culture or genotype background variation. The result showed that the AacCas12b‐induced mutations (on‐target editing) are solely present in edited plants, not in control plant. In the PC plant, there have some SNPs（base substitutions）mutations, possibly due to somaclonal variation. In total, 58 122, 104 841, 79 024 and 53 386 unique indels, and 150 917, 190 775, 149 747 and 170 704 unique SNPs were detected in a158, a297, b133 and b157 plants, respectively (Figure 3b). 11 322 SNPs and 9478 indels existed in a158, a297, b133 and b157 plants (Figure 3b). After filtering the shared variations, the remaining individual variations (48 644, 95 363, 69 546, 43 908 indels and 139 595, 179 453, 138 425, 159 382 SNPs in a158, a297, b133 and b157 lines, respectively) were filtered for further detection of AacCas12b‐induced off‐target mutations (Figure 3b). These variations were mapped into with the 1864 and 1490 predicted potential off‐target sites at sgRNA1 and sgRNA2 sites by Cas‐OFFinder to see whether they are overlap. The result exhibited there has no bona fide off‐target mutations were detected at these potential off‐target sites because the WGS detected variations in the AacCas12b edited plants were not overlapped with the predicated off‐target sites. Therefore, the WGS data suggest that AacCas12b does not cause detectable off‐target mutations and so is highly precise in cotton genome editing.

**Table 3:** Indels and SNPs of Cas 12b edited cotton revealed by whole‐genome sequencing

Lines	Plants vs Ref		Plants vs Ref/WT/PC		Individual variations	
	Total_Indel	Total_SNP	Unique_Indel	Unique_SNP	Individual Indel	Individual SNP
a158	1 773 469	3 237 186	58 122	150 917	48 644	139 595
a297	1 772 644	3 237 274	104 841	190 775	95 363	179 453
b133	1 772 789	3 237 410	79 024	149 747	69 546	138 425
b157	1 773 068	3 237 395	53 386	170 704	43 908	159 382
PC	1 773 889	3 237 136	–	–	–	–
WT*	1 774 432	3 237 453	–	–	–	–

The ‘Plants vs Ref’ represents the high confidence variations of per plant compared with TM‐1 reference genome. The ‘Plants vs Ref/WT/PC’ represents the variations of per transgenic‐edited plants compared with TM‐1, WT and PC. The individual variations indicated that edited plants have the similar genotype as PC plants, but differ from each other and contain specific variations. The ‘*’ indicating the genome data from these samples were cited from our previous report (Qin *et al*., 2020).

Overall, the manipulation of cotton plants using AacCas12b has been successfully established with no off‐target effects. Mutations were passed to the next generation where new editing events may occur in the offspring. This system is ideal for plant species that can tolerate temperatures above 40 °C, such as cotton that can grow well at temperatures reaching 45 °C. This result opens the way for further use of Cas12b in genome editing in plant species.

## Experimental procedures

### Vector construction

In this study, the pRGEB32‐AacCas12b‐GhCLA vector was modified from the CRISPR‐Cas9 vector pRGEB32‐GhU6.7 previously used for cotton genome editing in our laboratory (Wang *et al*., 2018). It contains a neomycin phosphotransferase (*NPTII*) selection marker, and sgRNA transcription was driven by cotton endogenous U6 promoter (GhU6‐7) with very high transcriptional activity. The plasmid pRGEB32‐GhU6.7 was digested by *Bstb*I and *Xba*I to remove the Cas9 sequence, which was replaced by Cas12b. The *AacCas12b* gene sequence was derived from NCBI PDB: 5WQE and synthesized by Nanjing Genscript Biotechnology Co., Ltd (Nanjing, Jiangsu, China). The AacCas12b unit was released from the template plasmid, and the product was inserted into the binary vector pRGEB32‐GhU6.7 using T4 ligation, generating the CRISPR/Cas12b vector pRGEB32‐AacCas12b (Figure 1a). For sgRNA construction, the tRNA‐sgRNA vector was synthesized and named pCTR as a template. *GhCLA* gene‐specific sgRNAs were designed in the exon region designated as sgRNA1 and sgRNA2 using CRISPR‐P online tool. pCTR was fused to the gene‐specific sgRNA cassettes by overlapping extension PCR. The PCR product then cloned into the *Bstb*Ⅰ‐digested pRGEB32‐AacCas12b vector.

### Agrobacterium‐mediated cotton transformation

The vector pRGEB32‐AacCas12b was introduced into *Agrobacterium* strain GV3101 via electroporation. Elite cotton (*Gossypium hirsutum*) cultivar Jin668 was used as the transformation receptor (Li *et al*., 2019c). Seeds were sterilized and cultured in a chamber without light for 6 days at 30 °C. Hypocotyls were cut into 5–10 mm cuttings and used as explants for *Agrobacterium*‐mediated transformation following our recently published methods (Sun *et al*., 2018; Wang *et al*., 2018).

### Heat treatment for the transgenic cells

After the explants co‐cultured with *Agrobacterium* for 2 days, the hypocotyl cutting was transferred to callus induction medium for 21 days, then these cuttings will produce some transgenic cells harbouring the *AacCas12b* gene and they were exposed to three different incubating temperatures of 42, 45 and 48 °C for 6 h, 12 h, 1 day, 2 days, 4 days, 7 days, 10 days, 12 days and 15 days. Two rounds of treatment were carried out, and the interval time for each treatment is one month.

### Mutation analysis of on‐target sites by Sanger sequencing

DNA was extracted from T0 transgenic cotton plants and wild type as control using a Plant Genome Extraction Kit (TIANGEN BIOTECH, Beijing, China). Specific primers for AacCas12b and sgRNA sequences were used to check confirm plant transformation. The targeted sites were amplified using site‐specific primers, and the amplicons were ligated in pGEMT‐Easy vector using T4 DNA ligase (Promega, Madison, USA). The vector was transformed into an *E. coli* strain using Top10, and positive clones were used for DNA Sanger sequencing.

### On‐target mutation detection by (Hi‐Tom) high‐throughput deep sequencing

Barcode‐based high‐throughput sequencing has been used for genotyping target genes in animals and plants (Loman *et al*., 2012). This approach was used to detect targeted editing efficiency. We used Hi‐TOM (high‐throughput tracking of mutations) sequencing which allows detection of 96 independent samples with a pair of unique barcodes for each (Liu *et al*., 2019). To identify editing events in Cas12b‐positive plants, a pair of 4 base combinations had been designed as the barcode tag for each sample. Each pair of markers was separately added to the 5′end of the forward and reverse primers to allow amplification of the target sequence. The corresponding barcode primers were used for PCR amplification of independent samples, and the resulting PCR products were mixed in equal amounts and purified (OMEGA kit, D2500‐02). The pooled DNA fragments were subjected to Illumina sequencing, and data were analyzed via the Hi‐TOM website (http://www.hi‐tom.net/hi‐tom/).

### The detection of off‐target mutation by whole‐genome sequencing (WGS)

Genomic DNA from four edited plants, one WT and one PC plant were extracted using the TIANGEN Plant Genomic DNA Kit (TIANGEN, Cat.#DP305‐03). The extracted DNA was sequenced using an Illumina NovaSeq sequencer at a sequencing depth of 50× coverage. To identify potential off‐target site edits, the BatMis and Cas‐OFFinder algorithms were used to compare the two sgRNA target sites of *GhCLA* against the TM‐1 reference genome (seed sequences ≤5 mismatches with the sgRNAs sequences) (Bae *et al*., 2014; Tennakoon *et al*., 2012; Wang *et al*., 2019). The CRISPResso algorithm was used with sequenced data to identify mutations at potential off‐target sites, including insertions, deletions and substitutions (Pinello *et al*., 2016). The PC plant was used as a positive control plant and was generated by tissue culture following transformation with the T‐DNA insertion and CRISPR‐Cas12b component, but without heat treatment. The genome of edited plants was compared with the genomes of WT plants and positive plants to filter out genotypic and somaclonal background variation following our previous report (Li *et al*., 2019b).

## Conflict of interest

The authors have declared that no competing interests exist.

## Author contributions

S.X.J. and X.L.Z. designed the project. Q.Q.W., F.Q.W., B.L., X.D., H.P.R., Y.J.L., Z.P.X., L.Q., L.S. and J.W.Z. performed experiments and wrote the manuscript. Q.Q.W. performed genotype data and W.G.S. analysed the data. S.X.J., J.Y.L., M.A. and K.L. revised the manuscript. All authors read and approved the final manuscript.
